# Maternal Death in Rural Ghana: A Case Study in the Upper East Region of Ghana

**DOI:** 10.3389/fpubh.2018.00101

**Published:** 2018-04-09

**Authors:** Paschal Awingura Apanga, John Koku Awoonor-Williams

**Affiliations:** ^1^Talensi District Hospital, Ghana Health Service, Talensi, Ghana; ^2^Policy, Planning, Monitoring and Evaluation Division, Ghana Health Service, Accra, Ghana

**Keywords:** maternal mortality, rural, strengthening, healthcare, antenatal care

## Abstract

Maternal mortality remains a challenge in providing quality maternal and other reproductive healthcare services in Ghana. This is a case investigation of a maternal death in rural Ghana that seeks to unravel the circumstances that lead to her death. We conducted three in-depth interviews with healthcare staff as well as a focused group discussion comprising of six relatives of the deceased, including her husband. The investigation revealed that lack of logistics, medical, and laboratory equipment, inadequate knowledge about the benefits of antenatal care services as well as non-adherence of healthcare workers to treatment protocols and standard operating procedures were found as major setbacks to the provision of effective and quality maternal healthcare services in Ghana. It is, therefore, imperative for the Government of Ghana and other Non-Governmental Organizations to invest in strengthening the healthcare delivery system especially in rural Ghana by making available basic logistics, medical, and laboratory equipment, as well as improving upon maternal health education, and consistently organizing capacity building training programs for healthcare workers.

## Introduction

Maternal mortality is defined as deaths occurring in women, while pregnant or within 42 days of termination of pregnancy irrespective of the duration and site of the pregnancy, from any cause related to or aggravated by the pregnancy or its management, but not from accidental or incidental causes (1). Maternal mortality continues to be a great concern with almost (99%) all maternal deaths occurring in developing countries with more than half in sub-Saharan Africa. 1 in 180 pregnant women die during childbirth when compared to 1 in 4,900 in developed countries. 75% of maternal deaths occur as a result of complications due to pregnancy and childbirth (2).

Ghana’s maternal mortality ratio declined from 760 per 100,000 live births in 1990 to 319 per 100,000 live births in 2015 (1, 2). The pace of decline in maternal mortality has been slow and this led to Ghana’s inability to achieve the millenium development goal target of 190 per 100,000 live births in 2015. The maternal mortality ratio remains high and requires strenuous efforts if Ghana has to achieve the sustainable development goal target of 70 per 100,000 live births in 2030. Most maternal deaths occur in the rural areas as compared to urban areas. This has largely been attributed to the high prevalence of skilled birth attendance of 74% in urban areas as compared to 43% in the rural areas (3–5). Several other factors have been implicated as major contributory factors to maternal deaths in Ghana. Low antenatal coverage, socio-cultural factors, lack of logistics, equipment, and blood at healthcare facilities has been largely blamed as reasons for high maternal mortality in Ghana (6–9). It was observed that maternal deaths are usually directly related to causes, such as hemorrhage, unsafe abortion, hypertensive disorders, infections, and obstructed labor while indirect causes, include malaria, HIV/AIDS, and anemia (6, 8). Other factors, including poverty, lack of skilled health personnel, and poor transport system have contributed to the high maternal mortality ratio in Ghana (7). This argument seems to be supported by the Thaddeus and Maine’s framework which posit that factors of this nature often result into one of the three delays; delay in making a decision to go to hospital, delay in arriving at the hospital, and a delay in getting treatment at a health facility which often lead to maternal mortality (10).

The healthcare system in Ghana is structured in five levels. The tertiary hospitals which are also teaching hospitals are at the apex of the healthcare delivery ladder. This is followed by the regional hospitals which provide specialist care for patients and also serve as referral points for district hospitals (11). The district hospital provides care at the district level and also serves as referral points for the sub-district facilities (facilities at the community level). The lowest facilities in the healthcare delivery ladder are the community clinics or community-based health planning and services (CHPS) (11). Although maternal mortality has been high in Ghana, several interventions have been implemented toward addressing the issue. The free maternal healthcare policy introduced has helped to reduce maternal mortality in the country; however, poverty prevented some women from accessing the free maternal healthcare (12). These women were unable to access the free maternal healthcare because they could not afford the cost of transport to the nearest health facility (13). Other interventions, such as the Ghana Essential Health Intervention Program has contributed in reducing maternal deaths by strengthening the CHPS, which makes healthcare more accessible to the people especially in rural communities (14). Although many other interventions have been implemented at national, regional, and community levels to reduce maternal mortality, its high ratio still remains a major concern in Ghana (15–19).

The upper east region (UER) of Ghana is not spared in this predicament. The 2013 annual report revealed that maternal mortality is a major public health issue as the region recorded 34 maternal deaths (20), hence, the occurrence of a maternal death on February 18, 2015 in a district hospital in the UER is of great concern. The decision to investigate this maternal death is due to the unique circumstances that led to the death of the woman. We believe that our findings will be very relevant in strengthening institutional, regional, and national policy, and decision making to improve upon maternal care services.

## Case Presentation

**Figure 1:**
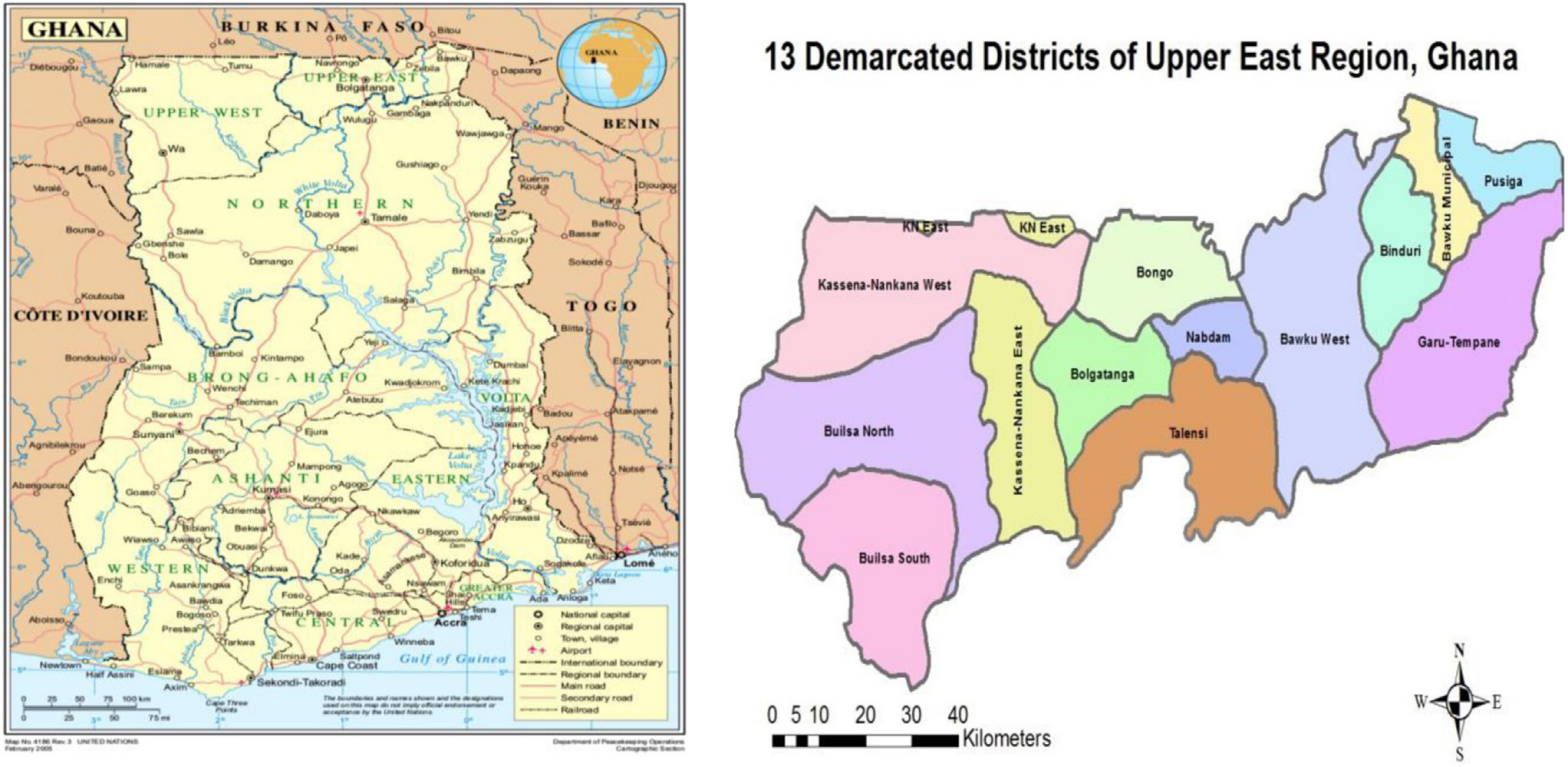
The study area in rural Ghana. Source: UN Department of Peacekeeping/Cartographic section and Map from UER Health Directorate.

This investigation was conducted from 18th to 24th February 2015 in a district in the UER of Ghana (Figure 1). Records at both a CHPS compound and a district hospital, where the client (now deceased) attended antenatal care (ANC) services and died were reviewed. The maternal health record booklet and patient folder notes of the deceased at both health facilities were reviewed. Records of the deceased were reviewed from 15th September, 2014, her first visit to ANC to the day of death, 18th February, 2015. In-depth interviews were conducted with the attending physician and the maternity unit midwife in charge of the district hospital. Also, in-depth interview was held with the midwife in charge of the CHPS compound, while a focused group discussion (FGD) made up of six relatives of the deceased, including her husband was held on events leading to her death. Enough time was allowed for the deceased to be buried before the FGD was conducted with the family. This was after the health team from the CHPS compound and the district hospital had sympathized with the family of the deceased in line with standard cultural practices within the Ghanaian context.

## Healthcare at the CHPS Compound

The investigation revealed that the deceased was G2 P1 (alive) with a gestation of 28 weeks. The deceased was 22 years old living in one of the communities in the UER. She was married, a Christian and unemployed. Records revealed her first attendant at ANC on September 15, 2014 at the CHPS compound, which is a community health facility, where the deceased lived, and this was followed by three more visits during which she had no complain of ill health. Records revealed that at 8:30 a.m. on February 17, 2015, the deceased presented to her community’s CHPS compound with complaint of general body pains, general weakness, and headache, a day before her death. There were no signs of diarrhea, vomiting, or any discharge or bleeding from her vagina. The disease also had no known history of chronic diseases like hypertension, diabetes, sickle cell, or asthma and was not on any medication at the time of reporting. A pelvic scan done at 16 weeks of her pregnancy was normal.

At the time of reporting and examination, her blood pressure was 60/40 mg, hemoglobin (Hb) concentration was 8.7 g/dl, fetal heart rate was present and was normal. However, her pulse and temperature were not taken. A malaria parasites test conducted was negative. The patient (deceased) was detained in the CHPS facility and was prescribed and given the following medication: 600 mg of oral quinine eight hourly for 7 days, oral folic acid 5 mg daily for 30 days and was administered intravenously 500 ml of normal saline. She was then discharged to go home the same day. There was neither record nor documentation of the time of her discharge from the facility.

The Ghana national policy on malaria diagnosis and treatment explicitly stated confirmation of malaria parasites before treatment. When questioned reason for the treatment given to the woman, this is what the midwife at the CHPS compound had to say:

Even though the malaria parasites test was negative, we cannot do any other test apart from looking for malaria parasites. I consulted with my colleague at work and we had to give quinine because the woman was very weak. I also gave normal saline because the blood pressure was low (midwife, IDI, CHPS compound)

## House of Deceased

In an interview, the husband of the deceased recounted what happened when they got home after discharge from the CHPS compound;

When we got home on the day of the discharge, she was very fine; the nurse even told her if she develops any complains she should return (Husband of deceased, FGD, community)

According to a relative of the deceased, the next day, February 18, 2015 at about 3:00 p.m., while at home the deceased experienced sudden abdominal pain and this was immediately followed by a spontaneous delivery of the fetus with profuse bleeding. This is what the deceased relative explained:

We realized she had delivered a small fresh child but dead, we had to clean her with different cloths as the blood was flowing non-stop. When we cleaned several times and the blood now stopped flowing, we realized she became very weak and restless. We now had to look for a motorbike to rush her to the district hospital because we knew the condition was above the community CHPS compound and it took us a long time, about two (2) hours to get a motorbike. We had to rush her to the hospital on the motorbike with the deceased sandwiched between the rider and a relative who supported her because she was restless (Relative of deceased, FGD, community)

## Healthcare at the District Hospital

The deceased was sent to the District Hospital as it is the primary referral facility for health facilities in the district. Information at the district hospital revealed that the patient (deceased) arrived at the district hospital at 6:10 p.m., where it was observed that she was very pale, gasping for breath with unrecordable blood pressure and pulse. The attending medical personnel quickly initiated resuscitation based on the severity of her clinical presentation with a liter of intravenous normal saline, at the same time called the national ambulance service, while a referral letter was written for relatives to send her to the next level of care which is the regional referral hospital. Among reason for the decision to refer to the regional hospital apart from the severity of her clinical presentation was the non-availability of oxygen and blood transfusion services at the district hospital. Medical personnel having to deal with emergency situations in the UER are faced with many practical challenges and these challenges largely determine the outcome of treatment. This is what the attending physician assistant had to say when questioned on what happened:

When I examined the patient I knew she needed oxygen and blood immediately to save her life. I had to urgently refer because I knew the patient needed oxygen and blood which we did not readily have at our facility, however, in the process of giving normal saline, unfortunately, the client passed away before the national ambulance could arrive. The cause of death was severe anemia and haemorrhagic shock due to postpartum hemorrhage (Physician Assistant, IDI, The District Hospital)

The midwife in charge of the district hospital also has this to say:

I had to immediately call the Physician Assistant to attend to her because the patient condition was critical, I have never experienced this case in our maternity unit before (midwife, IDI, The District Hospital)

Against all medical advice and importance of an autopsy, the relative of the deceased took the corpse away citing tradition that does not allow an autopsy conducted on a deceased that died through pregnancy. In an interview with the deceased husband and relatives to further understand what happened prior to arrival at the district hospital, the husband of the deceased stated:

My wife was not on any medication apart from what was given to her at the CHPS compound. Throughout this pregnancy, she was never sick except this her current predicament (Husband of deceased, FGD, Community)

Clearly showing her frustration at the situation the family found themselves in, this is what a relative of the deceased had to say:

You people (healthcare workers) need to educate the women on the importance of ANC in this community if you have to promote quality healthcare in the community because some of them (community) don’t know they have to immediately report to the hospital when they notice even the slightest bleeding in pregnancy (Another relative of deceased, FGD, Community)

In the same interview, the husband of the deceased stated:

I agree with my brother because when my wife was pregnant, I quarreled with her on one occasion when she missed out on one of the antenatal visits as I did not see any writing on her maternal health record book, so she lied to me that she went for ANC but that healthcare workers did not have a pen to write on it. I think you people (healthcare workers) have to emphasize on the benefits of ANC to our women (Husband of deceased, FGD, Community)

## Discussion

The investigation revealed that some healthcare workers (HCWs) still sort to treatment of malaria when test results are negative. Although this contradicts the recommended guidelines for treatment of malaria in Ghana (21), it was found to be consistent with other studies observed that treatment of malaria was sometimes administered in spite of negative diagnosis in Ghana (22). The Ghana’s national malaria policy does not recommend on the use of oral quinine as the first drug of choice for uncomplicated or severe malaria in third trimester of pregnancy (21, 23). This might be due to the poorly resourced nature of health facilities in rural communities in northern Ghana which tends to make it difficult for them to carry out basic and ordinary medical and laboratory tests as they lack the requisite equipment and logistics (22). Failure to carry out such basic test makes it difficult to rule out other possible existing infections that could have caused hypotension and stillbirth in pregnancy (24, 25). Although intravenous normal saline was given at the CHPS compound in an attempt to revert the situation, it was also not clear what level of blood pressure was achieved before discharge of the deceased, since there was no documentation. This calls for the need for better documentation by HCWs if obstetric care has to be improved (26).

The delay in seeking medical care is a major contributory factor that led to her death after the patient had profuse bleeding per vaginam after spontaneous delivery of the fetus. This was worsened by a further delay in reaching the health facility as means of transport to carry her was immediately not available, leading to the family spending several hours to secure a motorbike. Although the delay in securing means of transport could have been attributed to the low socioeconomic status of the deceased family, they should have reported back for treatment at the CHPS compound which was near their home (about 400 m). This could have possibly prevented the delays as the CHPS compound would have called the district hospital for an ambulance for a referral. Studies have reported that such delays are the major causes of maternal mortality in developing countries (27). It is, therefore, paramount for key messages at ANC services and educational campaigns to continuously emphasize on the need for pregnant women not to delay at home when they are sick. The implementation of a mobile-phone system at community level could contribute in improving emergency obstetric and neonatal care as it will provide an interactive platform between the community health worker and the pregnant woman (28). Other studies have suggested that birth preparedness and complication readiness which is a key component of globally accepted safe motherhood programs could be implemented in rural communities to reduce delays which women experience during obstetric complications as it addresses issues with transport when they need healthcare (29).

A hemoglobin level of 8.7 g/dl recorded by the deceased at the CHPS compound was a case of moderate anemia (22), and moderate anemia has been linked as an indirect cause of maternal mortality and stillbirth (30, 31). Additionally, the absence of oxygen and readily available blood at the district hospital has exposed how weak rural healthcare services can be. It is most likely that if oxygen and blood were readily available, the deceased’s life could have possibly been saved. Several studies have recommended that healthcare facilities in rural settings needs to be equipped with the necessary logistics, equipment, and ambulances if maternal mortality is to combate effectively and efficiently (32, 33).

## Conclusion

This investigation has shown how poorly resourced healthcare systems in developing countries like rural Ghana affect the provision of essential maternal healthcare services. It is important for Governments and other Non-Governmental Organizations interested in health to make healthcare system strengthening a priority especially in rural settings if the war against maternal mortality is to be won. Lessons from this study revealed that primary healthcare hospitals that act as primary referral facilities should be equipped with the necessary logistics and equipment to provide quality maternal care services. HCWs at the community level especially staff of CHPS compounds should be trained on how to recognize danger signs in pregnancy. Additionally, HCWs should be reminded and monitored regularly to manage pregnancy-related complications within their capacity, while cases beyond them immediately referred to the appropriate healthcare facility. More so, the Ghana Health Service should intensify educational campaigns on the benefits of ANC and the need for all pregnant women to take ANC services seriously. It is also important for HCWs to adhere to standard treatment protocols and standard operating procedures at work to help promote quality maternal care services, including ANC services.

## Limitations of the Study

Retrieval of the deceased patient folder notes was difficult. Secondly, the refusal of relatives of the deceased to allow an autopsy performed on the deceased was a setback and limitation as we could not determine the pathological cause of death.

## Ethics Statement

Approval was given by the Upper East Regional Health Directorate. Consent and approval was also given by the deceased family for the study and publication. Participants were well informed about the study and right to withdraw from the study even after participation.

## Author Contributions

PA conceived the study. PA and JA-W designed the study and collected the data. PA and JA-W analyzed the data and wrote the draft. Both authors read and approved the final draft.

## Conflict of Interest Statement

The authors declare that the research was conducted in the absence of any commercial or financial relationships that could be construed as a potential conflict of interest.

## Abbreviations

ANC, antenatal care; BPACR, birth preparedness and complication readiness; CHPS, community-based health planning and services; FGD, focused group discussion; GEHIP, Ghana essential health intervention program; HCWs, healthcare workers; IDIs, in-depth interviews; NGOs, non-governmental organizations; SOPs, standard operating procedures; UER, upper east region.
